# Uptake and impact of COVID-19 preventive measures amongst migrant populations in the Netherlands: Marieke Torensma

**DOI:** 10.1093/eurpub/ckac131.047

**Published:** 2022-10-25

**Authors:** M Torensma, J Harting, L Boateng, C Agyemang, Y Lassooy-Tekle, Y Jacob, M van den Muijsenbergh, F el Fakiri, M Prins, K Stronks

**Affiliations:** 1 Public and Occupational Health, Amsterdam UMC, Location AMC, Amsterdam, Netherlands; 2 Amsterdam Public Health Research Institute, Amsterdam, Netherlands; 3 Primary and Community Care, Radboud University Medical Centre, Nijmegen, Netherlands; 4 Pharos Expertise Centre on Health Disparities, Utrecht, Netherlands; 5 Epidemiology, Health Promotion and Care Innovation, Public Health Service of Amsterdam, Amsterdam, Netherlands; 6 Infectious Diseases, Public Health Service of Amsterdam, Amsterdam, Netherlands; 7 Infectious Diseases, Amsterdam UMC, Location AMC, Amsterdam, Netherlands; 8 Cultuur in Harmonie, Zeewolde, Netherlands

## Abstract

Uptake of preventive measures to reduce transmission of viruses such as SARS-CoV-2, is crucial in the control of pandemics. To ensure equitable uptake we explored contextual factors that shaped uptake of COVID-19 preventive measures amongst smaller, albeit substantial, migrant populations in the Netherlands. 39 persons of Eritrean, Ghanaian, Indonesian and Filipino origin, with diverse legal status and length of stay in the Netherlands, participated in five online focus group discussions. Thematic analysis of data was informed by concepts from the Precaution Adoption Process Model and Protection Motivation Theory. Awareness and knowledge of preventive measures was shaped by limited Dutch proficiency, access to understandable information and interference of misinformation. Engagement by preventive measures was subject to COVID-19 threat appraisal and the ease with which complex behavioural messages could be translated to individual situations. Perceived vulnerability of undocumented migrants in particular, motivated information-seeking. A strong social norm to keep with cultural and religious practices, and limited opportunity for preventive behaviour in work and home context hindered uptake of preventive behaviour. Preventive measures brought about job, food, and housing insecurity, and increased barriers in access to healthcare for undocumented migrants. Migration-related, sociocultural, and socioeconomic factors shape uptake of preventive measures. Preventive measures negatively impact work, housing and access to healthcare of undocumented migrants. Our results suggest importance of multilingual information tailored to literacy needs; education and modelling of behaviour; and, regulations to ensure continued access to financial and material resources to minimise negative spill-over effects. Results were incorporated in two policy briefs advising local and national government. Collaboration with municipal health services lead to multilingual public health information.

**Key messages:**

• Migration-related, sociocultural, and socioeconomic factors shape uptake of preventive measures.

• Preventive measures negatively impact work, housing and access to healthcare of undocumented migrants.

